# Supplementary material for: WordCommentsAnalyzer: A windows software tool for qualitative research
Source: F1000Res. 2018 Sep 4;7:536. Originally published 2018 May 3. [Version 2] doi: 10.12688/f1000research.14819.2 (PMC6137411; doi:10.12688/f1000research.14819.2)
Supplement: Supplementary file 3 [file f1000research-7-17614-s0002.tgz › b2a79496-4ada-47c4-9682-a99ce867836d.zip!/Eight Iranian Health Journals/Journal of Research in Rehabilitation Sciences _11.docx]

© Journal of Research in Rehabilitation Sciences . This work is licensed under CC BY-NC 4.0 (https://creativecommons.org/licenses/by-nc/4.0/).

Volume & Issue: دوره 11، شماره 6: 1394

Title: بررسی تأثیر تقارن در ابتلا به استئوآرتریت متوسط زانو بر گزارش بیمار از درد و عملکرد: مطالعه مقدماتی (قسمت اول)؛ مقیاس درد مداوم و دوره‌ای استئوآرتریت زانو

http://jrrs.mui.ac.ir/index.php/jrrs/article/view/2552

مقدمه: استئوآرتریت زانو شایع‌ترین نوع التهاب مفصل می‌باشد که به دلیل درد و آسیب ساختارهای مفصل، به تدریج باعث محدودیت عملکرد فرد می‌شود. هدف از انجام مطالعه حاضر، مقایسه گزارش درد در مقیاس درد متناوب و مداوم استئوآرتریت توسط افراد مبتلا به استئوآرتریت متوسط متقارن و نامتقارن بود. مواد و روش‌ها: با بررسی معیارهای ورود و خروج، از میان 20 فرد مبتلا به استئوآرتریت متوسط زانو، دو گروه متقارن و نامتقارن زانو به صورت هدفمند تشکیل داده شد. اطلاعات جمعیت‌شناسی همه شرکت کنندگان پس از اخذ رضایت‌نامه آگاهانه جمع‌آوری گردید و گزارش افراد در مقیاس درد متناوب و مداوم زانو ثبت شد. پاسخ افراد به هر سؤال و نمره فرد در هر بخش پرسش‌نامه و در کل در دو گروه مورد مقایسه قرار گرفت. نتایج در دو گروه با استفاده از آزمون Mann–Whitney تجزیه و تحلیل گردید. یافته‌ها: دو گروه تفاوت معنی‌داری در نمره درد مداوم، درد متناوب و کل نشان ندادند (05/0 < P). هر چند در مقایسه پاسخ گروه‌ها به هر سؤال مشخص شد که در گروه نامتقارن، هر دو نوع درد مداوم (04/0 = P) و درد متناوب (دردی که می‌آید و می‌رود) (02/0 = P) تأثیر منفی بیشتری بر خواب داشت و شدت درد متناوب بیشتر بود (04/0 = P). نتیجه‎گیری: به نظر می‌رسد که با وجود عدم تفاوت معنی‌دار در انواع درد ناشی از استئوآرتریت زانو، شکایت این بیماران از شدت درد و تأثیر آن بر کیفیت خواب متفاوت است.

Title: بررسی تأثیر تقارن در ابتلا به اسـتئوآرتریت متوسط زانو بر گزارش بیمار از درد و عملکرد: مطالعه مقدماتی (قسمت دوم)؛ مقیاس پیامدهای ضایعات و استئوآرتریت زانو

http://jrrs.mui.ac.ir/index.php/jrrs/article/view/2550

مقدمه: استئوآرتریت زانو یک بیماری شایع وابسته به سن می‌باشد. هدف از انجام مطالعه حاضر، تعیین تأثیر تقارن در درگیری زانوهای افراد مبتلا به استئوآرتریت متوسط زانو بر گزارش شخصی آنان از شدت علایم، میزان درد، عملکرد در فعالیت‌های مختلف و کیفیت زندگی بر اساس پرسش‌نامه KOOS (Knee Injury and Osteoarthritis Outcome Score) بود. مواد و روش‌ها: 20 فرد مبتلا به استئوآرتریت متوسط زانو، به صورت هدفمند در دو گروه افراد مبتلا به درگیری متقارن و افراد مبتلا به درگیری نامتقارن زانو تقسیم شدند. پس از اخذ رضایت‌نامه آگاهانه، اطلاعات جمعیت‌شناسی افراد در فرم مخصوص ثبت گردید. سپس از افراد درخواست شد که به مقیاس پيامدهاي ضایعات و استئوآرتريت زانو پاسخ دهند. پاسخ افراد به بخش‌های مختلف پرسش‌نامه به صورت سؤال به سؤال و نیز در قالب امتیاز هر بخش در دو گروه مورد مقایسه قرار گرفت. پاسخ دو گروه از طریق آزمون Mann–Whitney تجزیه و تحلیل گردید. یافته‌ها: شدت درد در بالا رفتن از پله (030/0 = P)، نشستن و دراز کشیدن (040/0 = P)، بروز مشکلات در حین سوار و پیاده شدن از اتومبیل (010/0 = P)، برخواستن از رختخواب (009/0 = P) و جابه‌جا شدن در رختخواب (020/0 = P)، برخواستن از حالت نشسته، پوشیدن جوراب و حمام کردن (050/0 = P) در گروه متقارن بیش از گروه نامتقارن بود. همچنین، این گروه از احساس عدم اطمینان بیشتری در زانوی خود نسبت به گروه نامتقارن شکایت داشتند (030/0 = P)؛ بدین ترتیب توانایی انجام فعالیت‌های روزانه و کیفیت زندگی در گروه متقارن به طور معنی‌داری کمتر از گروه نامتقارن بود (040/0 = P) و (020/0 = P). نتیجه‎گیری: به نظر می‌رسد توانایی انجام فعالیت‌های روزانه و نیز کیفیت زندگی در افراد گروه متقارن به طور معنی‌داری کمتر از گروه نامتقارن باشد. انجام مطالعات بیشتر به منظور شناخت بهتر مشکلات عملکردی این افراد در زندگی روزمره توصیه می‌شود.

Title: اثر زمینه بینایی بر ظهور مهارت ویژه در پرتاب آزاد بسکتبال بازیکنان ماهر و کم‌تجربه

http://jrrs.mui.ac.ir/index.php/jrrs/article/view/2546

مقدمه: هنگامی ‌که مهارتی بر اثر تمرین بسیار زیاد، به‌ طور چشمگیری بهتر از مهارت‌های مشابه همان طبقه اجرا گردد، آن را مهارت ویژه می‌نامند. هدف از انجام پژوهش حاضر، بررسی تأثیر زمینه بینایی بر ظهور مهارت ویژه در پرتاب آزاد بسکتبال میان بازیکنان ماهر و کم‌تجربه بود. مواد و روش‌ها: 10 نفر از بازیکنان ماهر و 10 نفر از بازیکنان کم‌تجربه بسکتبال، ۱۵۰ شوت ثابت را از ۵ فاصله مختلف نسبت به حلقه پرتاب کردند. بازیکنان پرتاب‌های خود را در سه شرایط بینایی (روشن، یک نقطه‌ای و تاریک) انجام دادند. داده‌ها با استفاده از آزمون‌های تحلیل رگرسیون خطی، Paired t و تحلیل رگرسیون منحنی مورد تجزیه و تحلیل قرار گرفت. یافته‌ها: بررسی تحلیل رگرسیون خطی با استفاده از داده‌های چهار فاصله، ارتباط منفی معنی‌داری را بین فاصله و صحت اجرا در گروه ماهر نشان داد (68/0- = r، 050/0 > P). یافته‌های حاصل از آزمون Paired t حاکی از آن بود که بازیکنان ماهر، دارای عملکرد بهتری در خط پرتاب آزاد در شرایط روشن و یک نقطه‌ای بودند (050/0 > P)؛ در حالی ‌که بازیکنان ماهر در شرایط تاریک، عملکرد بهتری در خط پرتاب آزاد نداشتند. بازیکنان کم‌تجربه تنها رابطه خطی با شیب منفی بین فواصل مختلف و امتیازات عملکرد را نشان دادند (41/0- = r، 050/0 > P). همچنین، یافته‌ها تحلیل رگرسیون منحنی داده‌های گروه ماهر را بهتر تبیین نمود (70/0- = r، 050/0 > P).نتیجه‎گیری: نتایج مطالعه نقش اطلاعات بینایی را در ظهور اثر مهارت ویژه در افراد ماهر نشان داد.

Title: بررسی ارتباط مشكلات رفتاري کودکان مبتلا به فلج مغزي 7 تا 13 ساله با افسردگي مادران: مطالعه مقطعی

http://jrrs.mui.ac.ir/index.php/jrrs/article/view/2556

مقدمه: کودکان مبتلا به فلج مغزی در کنار مشکلات جسمی متعدد، دارای مشکلات روانی- اجتماعی همچون مشکلات رفتاری می‌باشند. داشتن کودک مبتلا به فلج مغزی خطر ایجاد افسردگی را در مادران افزایش می‌دهد. این در حالی است که مادر، مهم‌ترين نقش را در پرورش كودك ایفا می‌نماید. از این‌رو، هدف از انجام پژوهش حاضر، بررسی ارتباط بین مشکلات رفتاری کودکان مبتلا به فلج مغزی و میزان افسردگی مادران بود. مواد و روش‌ها: در این مطالعه، 76 کودک مبتلا به فلج مغزی 7 تا 13 ساله و مادران آن‌ها مورد بررسی قرار گرفتند. برای ارزیابی ارتباط مشکلات رفتاری در کودکان و سطح افسردگی مادران به ترتیب از پرسش‌نامه‌های ارزشیابی معلم Rutter و افسردگی Beck استفاده گردید. داده‌ها در نرم‌افزار SPSS مورد تجزیه و تحلیل قرار گرفت. یافته‌ها: 7/67 درصد از کودکان مبتلا به فلج مغزی دارای مشکلات رفتاری بودند و بین مشکلات رفتاری این کودکان و میزان افسردگی مادران ارتباط معنی‌داری وجود نداشت (49/0 = P، 08/0- = r). نتیجه‎گیری: از نتایج پژوهش حاضر چنین نتیجه‌گیری می‌شود که مشکلات رفتاری کودکان مبتلا به فلج مغزی نمی‌تواند عاملی برای ایجاد افسردگی در مادران تلقی گردد.

Title: تأثير پد متاتارسال بر فشار کف پایی ناحيه سرپنجه افراد مبتلا بهت نوروپاتي محیطی ناشی از دیابت: کارآزمایی بالینی متقاطع تصادفی

http://jrrs.mui.ac.ir/index.php/jrrs/article/view/2561

مقدمه: یکی از روش‌های کاهش فشار بر سطح کف پا جهت جلوگیری از زخم ناشی از دیابت، استفاده از کفی طبی است. هدف از انجام مطالعه حاضر، بررسی اثرات استفاده از ساپورت قوس عرضی پا (پد متاتارسال) بر فشارهای وارد بر ناحیه پنجه پا بود. مواد و روش‌ها: در این کارآزمایی بالینی متقاطع تصادفی، 18 داوطلب (5 زن و 13 مرد) دچار نوروپاتی محیطی ناشی از دیابت شرکت کردند. فشار وارد آمده بر کف پای افراد در حین راه رفتن، با استفاده از یک صفحه ثبت فشار در سه حالت تصادفی پای برهنه، دارونما و پد متاتارسال اندازه‌گیری شد. داده‌ها با استفاده از آزمون تحليل واريانس با اندازه‌هاي مكرر تجزیه و تحلیل گردید. یافته‌ها: پد متاتارس در مقایسه با پای‌ برهنه و دارونما، باعث کاهش معنی‌دار میانگین فشار وارد آمده بر پنجه در فاز استانس راه رفتن شد (001/0 > P). همچنین، پد متاتارس در مقایسه با پای‌ برهنه و دارونما، میزان انتگرال نیرو- زمان را در ناحیه پنجه به طور معنی‌داری کاهش داد (001/0 > P). اختلاف معنی‌داری در اندازه انتگرال فشار- زمان کل سطح کف پا بین حالات مختلف مشاهده نشد. نتیجه‎گیری: پد متاتارس می‌تواند از طریق انتقال فشار به نواحی دیگر (میدفوت)، میزان فشار وارد آمده بر پنجه را کاهش دهد. به نظر می‌رسد که استفاده از برجستگی پد متاتارسال بتواند در پیشگیری از زخم پنجه پای افراد مبتلا به دیابت مؤثر باشد.

Title: خودمردم‌نگاري، شيوه پژوهشي نوين در توان‌بخشي و سلامت (بخش دوم)

http://jrrs.mui.ac.ir/index.php/jrrs/article/view/2402

مقدمه: هدف از انجام پژوهش حاضر، تجزيه و تحليل تجارب زندگي پژوهشگر از زندگي خود با محدوديت بينايي به شيوه خودمردم‌نگاري بود. مواد و روش‌ها: داده‌هاي مطالعه بر اساس روايت‌هاي زندگي نويسنده دوم جمع‌آوري شد و طي مصاحبه و يا در فرصت شخصي روايتگر، بر روي فايل‌هاي صوتي بازگو گردید. اين داده‌ها توسط نويسنده اول با روش تحليل درون‌مایه مورد تجزيه و تحليل قرار گرفت. یافته‌ها: داده‌ها به صورت دو درون‌مایه اصلي «موانع و منابع تاب‌آوري» به همراه 64 كد دسته‌بندي گردید. نتیجه‎گیری: فرد دارای محدودیت نابینایی، ممکن است در مسیر زندگی خود تاب‌آوری سطح بالایی را نشان دهد و مانند سایر افراد به موفقیت‌های تحصیلی، شغلی و اجتماعی دست پیدا کند، اما برای قرار گرفتن در مسیر تاب‌آوری، لازم است بر موانع ممکن غلبه کند و منابعش را تقویت نماید.

Title: خودمردم‌نگاري، شيوه پژوهشي نوين در توان‌بخشي و سلامت (بخش اول)

http://jrrs.mui.ac.ir/index.php/jrrs/article/view/2558

خودمردم‌نگاری شیوه‌ای پژوهشی است که مفهوم «خود» را در یک بافت اجتماعی مورد تجزیه و تحلیل قرار می‌دهد. این شیوه که به طور سنتی در پژوهش‌های علوم اجتماعی مورد استفاده قرار می‌گیرد، به تازگی در حیطه سلامت نیز جای خود را باز کرده است. هدف از انجام پژوهش حاضر، معرفی روش پژوهش خودمردم‌نگاري و كاربرد آن در پژوهش‌هاي توان‌بخشي و سلامت بود. جهت معرفي اين شيوه، مروري بر تعاريف،‌ انواع، فرايند اجرا، مسايل اخلاقي و روش نگارش يافته‌هاي خودمردم‌نگارانه با نگاهي بر مقالات چهره‌هاي شاخص در اين زمينه انجام گرفت. خودمردم‌نگاري نوعي پژوهش كيفي است كه تجارب شخصي را به فرهنگ عمومي پيوند مي‌زند. متخصصاني كه داراي نوعي محدوديت و ناتواني هستند، با استفاده از اين شيوه مي‌توانند جامعه بزرگ‌تر را از نيازهايشان آگاه سازند و از اين طريق، مدافعي براي برنامه‌هاي توان‌بخشي مناسب با گروه خاصي كه به آن تعلق دارند، باشند. مرور مقالات نشان داد كه توجه به نظر تخصصی فردی که دارای نوعي از محدودیت است، ارزش بسزایی برای سایر متخصصان دخیل در امر سلامت و توان‌بخشی دارد.

Title: کاربرد چارچوب طبقه‌بندی بین‌المللی عملکرد، ناتوانی و سلامت (ICF) در مداخله برای اختلالات گفتار و زبان کودکان پیش‌دبستانی: یک مرور نظام‌مند

http://jrrs.mui.ac.ir/index.php/jrrs/article/view/2420

مقدمه: با توجه به کمبود دانش مورد نیاز درباره چارچوب مفهومی ICF (International Classification of Functioning, Disability and Health) در ایران، مطالعه حاضر با مرور هدفمندی بر مقالات منتشر شده در زمینه به کارگیری ICF در حوزه اختلالات گفتار و زبان کودکان پیش‌دبستانی، سعی بر آن داشت که آسیب‌شناسان گفتار و زبان را با این چارچوب و سطح کیفی مطالعاتی که تاکنون در این حوزه انجام شده است، آشنا کند. مواد و روش‌ها: عبارت‌های «ICF و اختلالات ‌ارتباطی، ICF و اختلالات زبانی، ICF و اختلالات گفتاری» به عنوان کلید واژه‌های جستجو در بازه زمانی سال‌های 2005 تا 2015 در پایگاه‌های اطلاعاتی PubMed و Web of Knowledge به عنوان جامع‌ترین پایگاه‌های موجود در حوزه پزشکی، توان‌بخشی و رشته‌های وابسته در نظر گرفته شدند. یافته‌ها: 6 مقاله از 96 مقاله یافت شده، واجد شرایط ورود به مطالعه تشخیص داده شد. همه مقالات تأکید داشتند که چارچوب مفهومی ICF با ارایه دید همه جانبه‌، مفهومی از اختلالات گفتار و زبان کودکان را فراهم می‌کند که می‌تواند تعامل منسجمی بین شواهد تجربی و مشاهدات بالینی به وجود آورد و همه موانع و تسهیل کننده‌های موجود در زندگی ارتباطی کودک را مورد بررسی قرار می‌دهد و در نهایت، ارزیابی و درمان اختلالات گفتار و زبان کودکان به صورت مؤثرتر و هدفمندتری انجام خواهد شد. در این مقالات از میان درون‌مایه‌های (حوزه‌) موجود در چارچوبICF ، 735 درون‌مایه مورد استفاده قرار گرفت که بیشترین آن‌ها مربوط به مجموعه فعالیت و مشارکت این چارچوب بود. نتیجه‎گیری: چارچوب ICF به کمک اجزای خود و تعامل بین آن‌ها، از تمرکزی که بیشتر درمانگران به صورت سنتی و پزشکی تنها بر ساختار و عملکرد اندام‌های بدن دارند، جلوگیری می‌کند و تمرکز همه جانبه‌ای بر روی عملکرد، فعالیت، مشارکت و عوامل محیطی و فردی کودک، دارد. مطالعات انجام شده بر به کارگیری چنین رویکرد جامعی جهت ارزیابی و درمان دقیق اختلالات گفتار و زبان کودکان تأکید نمود. بر اساس نتایج به دست آمده از تعیین کیفیت مقالات چاپ‌ شده در این زمینه، اکثر مقالات مربوط به این موضوع به طور کلی در دنیا، در قالب غیر تجربی و حتی غیر پژوهشی چاپ شده‌اند و مطالعات تجربی بسیار کمی در این زمینه صورت گرفته است که در بهترین حالت، از سطح مطالعات توصیفی بالاتر نرفته‌اند.
